# CAR-Based Approaches to Cutaneous T-Cell Lymphoma

**DOI:** 10.3389/fonc.2019.00259

**Published:** 2019-04-16

**Authors:** Irene Scarfò, Matthew J. Frigault, Marcela V. Maus

**Affiliations:** ^1^Cellular Immunotherapy Program, Massachusetts General Hospital Cancer Center, Charlestown, MA, United States; ^2^Harvard Medical School, Boston, MA, United States; ^3^Broad Institute of Harvard and MIT, Cambridge, MA, United States

**Keywords:** T cell lymphomas/leukemias, CAR T cells, adoptive cell therapy, immunotherapy, cutaneous

## Abstract

Cutaneous T cell lymphomas (CTCL) are a heterogeneous group of malignancies characterized by the expansion of a malignant T cell clone. Chimeric Antigen Receptor (CAR) T cell therapy has shown impressive results for the treatment of B-cell tumors, but several challenges have prevented this approach in the context of T cell lymphoma. These challenges include the possibilities of fratricide due to shared T-cell antigens, T cell immunodeficiency, and CAR transduction of malignant cells if CAR T are manufactured in the autologous setting. In this review, we discuss these and other challenges in detail and summarize the approaches currently in development to overcome these challenges and offer cellular targeting of T cell lymphomas.

## Introduction

Cutaneous T-cell lymphomas (CTCL) are a heterogeneous group of malignancies of T-cell origin that occur primarily in the skin. They are the second most common form of extranodal Non-Hodgkin lymphomas (NHL) and their incidence has been increasing over the time (1). Mycosis fungoides (MF) and Sézary syndrome (SS) are the most common subtypes, which represent about 70–75% of CTCL (2, 3); other frequent types include primary cutaneous CD30+ T-cell lymphoproliferative disorders, adult T-cell leukemia/lymphoma and a portion of peripheral T-cell lymphoma not otherwise specified (PTCL-NOS). Although both cutaneous and/or systemic therapies have been developed for these tumors, long-term outcomes are characterized by high relapse rates with advanced forms of CTCL considered incurable. Recent progress has been made using immunotherapy approaches. These include the development of monoclonal antibodies, such as brentuximab vedotin, which has shown clinical efficacy leading to FDA approval in certain disease sub-types (4–6). Here, we discuss recent advances in adoptive Chimeric Antigen Receptor (CAR) T cell therapy and summarize the challenges and opportunities for the treatment of CTCL.

## Hurdles in the Development of Adoptive Cell Therapy for the Treatment of CTCL

**Figure 1:**
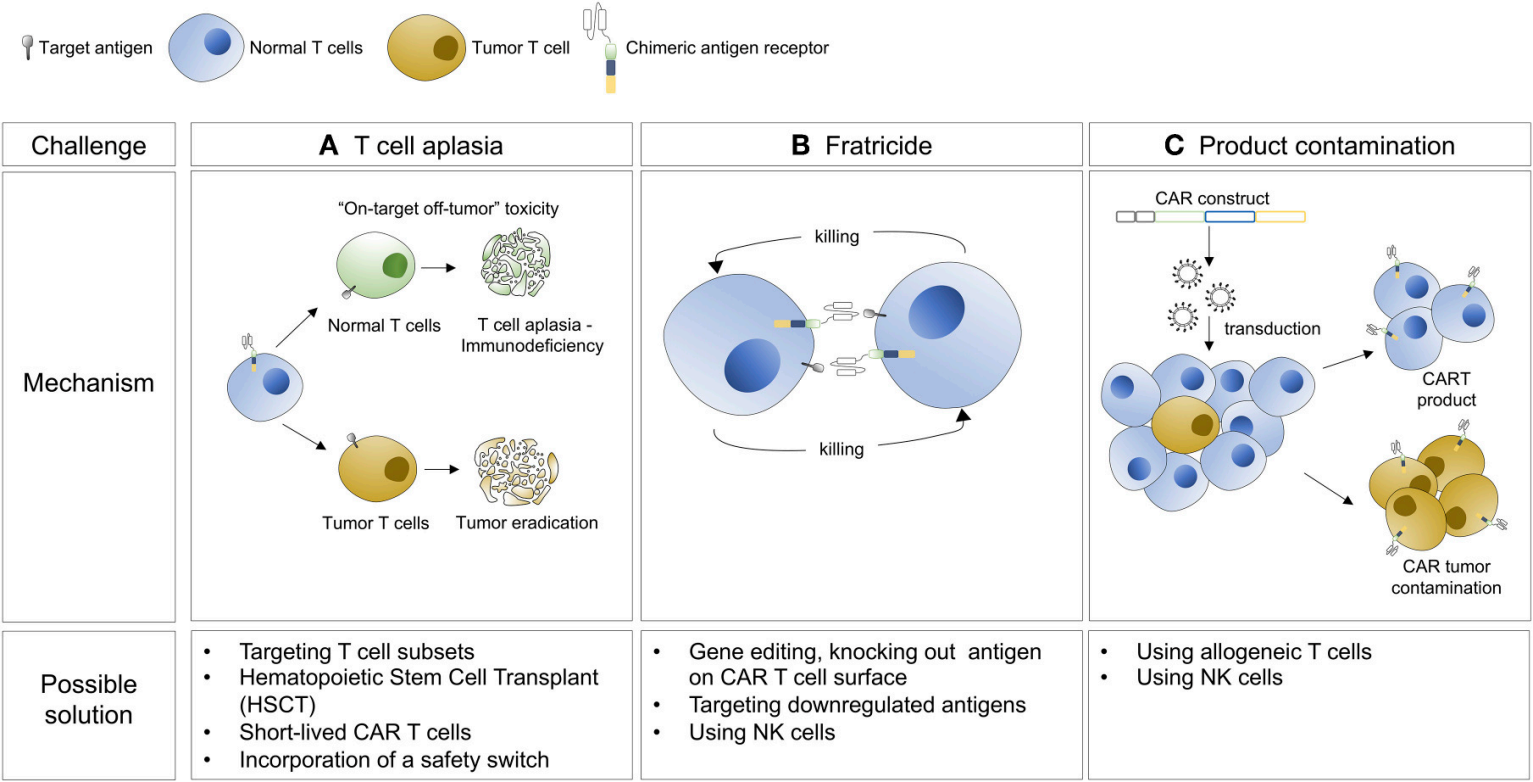
Hurdles associated with the development of CAR T cell therapy for the treatment of CTCL and possible solutions.

Immunotherapy has emerged as groundbreaking approach for the treatment of cancer and includes monoclonal antibodies, immune checkpoint blockade, tumor vaccines, and most recently, CAR T cell therapy. CAR-T cell therapy redirects a poly-clonal T-cell population against a tumor specific antigen in an MHC independent fashion. Based on impressive phase I/II data, CAR-T cell therapies are now approved for CD19+ B-cell malignancies including pediatric acute lymphoblastic leukemia (B-ALL) and large cell lymphoma (7, 8). Although B-cell aplasia is a common and manageable side effect of CD19 directed CAR-T cell therapy, T-cell aplasia is less tolerable in the long term, and can be life-threatening if it is not reversed or ameliorated. Furthermore, in the case of CTCL, malignant and normal T cells express many shared surface markers, limiting our ability to differentially target these populations. For this reason, special considerations have to be made when designing CARs for the treatment of T-cell lineage malignancies. First, permanent T cell aplasia is not acceptable, but approaches using either transient CAR T cell expression or persistence or suicide genes could be used to eliminate the CAR cells and allow for T cell immune reconstitution (Figure 1A). Second, killing of CAR-expressing cells by each other, known as fratricide, can undermine the *ex/in vivo* expansion of modified T cells and the generation of CAR T cell products (Figure 1B). Third, circulating tumor cells can contaminate leukapheresis products and be transduced with CARs during manufacturing, which could be associated with a growth advantage for the transduced tumor cells or resistance to CAR-T cell mediated cytotoxicity (Figure 1C). This phenomenon has been recently documented in a B-ALL patient relapsed after CTL019 treatment (9), whereby transduction of the tumor cells with the CAR led to “masking” the expression of the CD19 target antigen and therefore resistance to the CAR T cell-mediated killing. All these aspects need to be considered for the development of CAR T cell therapy against CTCL. However, the unmet need in T cell lymphomas is great, and effective treatments would represent a significant therapeutic advance.

## CAR T Cells Against T Cell Antigens

It has been difficult to identify targets uniquely expressed on malignant but not on normal T cells. One strategy has been to target molecules expressed by a subpopulation of T cells, or which are downregulated when T cells are activated. This approach has been adopted for the design of CAR against CD4, CD5, CD7, CD30, CD37, CCR4, and the 2 alleles of the T cell receptor beta chains (TRBC1/TRBC2) (Table 1).

**Table 1:** CAR T/NK cells for the treatment of CTCL.

	**Target antigen**	**CAR construct**	**Modifications**	**Models**	**Clinical trials**	**References**
CAR T cells	CD4	αCD4 CD28 41BB CAR	–	*in vitro*: KARPAS-299; Sézary syndrome, PTCL *in vivo*: KARPAS-299	–	(10)
	CD5	αCD5 CD28 CAR	–	*in vitro*: MOLT-4, CCRF-CEM, Jurkat, HuT78, SUP-T1; T-ALL *in vivo*: Jurkat	–	(11)
		αCD5 41BB CAR	–	*in vitro*: CCRF-CEM, Jurkat *in vivo*: Jurkat	–	(12)
	CD7	αCD7 CD28 CAR	CD7 CRISPR/Cas9 KO	*in vitro*: MOLT-4, CCRF, Jurkat, HuT78, SUP-T1; T-ALL *in vivo*: CCRF-CEM	NCT03690011	(13)
		αCD7 41BB CAR	CD7 protein expression blocker (PEBL)	*in vitro*: MOLT-4, CCRF-CEM, Jurkat, Loucy, KG1a *in vivo:* CCRF-CEM; ETP-ALL PDX		(14)
		αCD7 C CD28 41BB AR	CD7, TRAC CRISPR/Cas9 KO	*in vitro*: MOLT-4, MOLT-3, HSB-2, T-ALL *in vivo*: CCRF-CEM; T-ALL PDX		(15)
	CD30	αCD30 CD28 CAR	–	*in vitro*: KARPAS-299, HDLM-2, L428, L540, KH-M2, L1236 *in vivo*: KARPAS-299	NCT03049449, NCT01316146, NCT02690545, NCT02917083	(16)
	CD37	αCD37 41BB CAR	–	*in vitro*: FEPD, HuT78, PTCL	–	(17)
	CCR4	αCCR4 41BB CAR	–	*in vitro*: HH, HuT78, ML, HuT102, JB-6, Karpas299, SUDHL-1, SR-786, SUP-M2, DEL, Mac-1, Mac-2A, Mac-2B *in vivo*: ATL41214	–	(18)
	TRBC1	αTRBC1 CD28-OX40 CAR	–	*in vitro*: T-PLL, PTCL-NOS, ATLL *in vivo*: Jurkat	NCT0359054	(19)
	TRBC2	αTRBC2 CD28-OX40 CAR	–	*in vitro*: Loucy, MOLT13, BE13 *in vivo*: Loucy	–	(20)
CAR NK cells	CD3	αCD3 41BB CD28 CAR	NK-92 cells	*in vitro*: KARPAS-299, CCRF-CEM, Jurkat; PTCL, Sézary syndrome *in vivo*: Jurkat	–	(21)
	CD4	αCD4 CD28 41BB CAR	NK-92 cells	*in vitro*: KARPAS-299, HL60, CCRF-CEM; Sézary syndrome, T-ALL *in vivo*: KARPS-299	–	(22)
	CD5	αCD5 41BB CD28 CAR	NK-92 cells	*in vitro*: MOLT-4, CCRF-CEM, Jurkat; T-ALL, Sézary syndrome *in vivo*: Jurkat	NCT03081910	(23)
	CD7	αCD7 CD28 41BB CAR	NK-92 cells		NCT02742727	

### CD4

CD4 was one of the first molecules selected as target for CAR T cell therapy design. It is highly and uniformly expressed by T cell lymphomas, including CTCL which are predominantly comprised of peripheral CD4 positive T cells. In 2016, Pinz and colleagues showed preclinical efficacy of anti-CD4 CAR T cells *in vitro* and in a xenograft mouse model of ALCL (10). Although this approach demonstrated the potential for CAR-T cells in ALCL, ongoing CD4 depletion could lead to a T cell immunodeficiency similar to that observed in the acquired immunodeficiency syndrome (AIDS) induced by the human immunodeficiency virus (HIV).

### CD5

CD5 is another highly expressed antigen on malignant T cells (24, 25). In normal mature T cells, it has a costimulatory role in synergy with CD28 and TCR/CD3 (26–28); previous studies have shown that its expression is post-transnationally regulated (29). Anti-CD5 CAR T cells have been tested in two configurations. The first, designed by Mamonkin et al. included CD28 as costimulatory domain and showed a transient fratricide and a limited bystander killing of normal T cells due indeed to surface downregulation of CD5 protein (11). These CAR T cells demonstrated preclinical activity *in vitro* against different TCL and T-ALL cell lines, including the HUT78 Sézary syndrome cells, but only partial clearance of T-ALL xenograft tumor, suggesting a lack of CAR-T cell persistence. For this reason, Mamonkin and colleagues designed a second version of the CAR using 4-1BB as costimulatory domain. Interestingly, they reported a higher fratricide when expanding 4-1BB CAR T cells compared to CD28 CAR T cells. The authors demonstrated that 4-1BB upregulates ICAM-1 molecule increasing the stability of the immunological synapse and consequent killing (12). In order to regulate CD5 targeted killing, the authors put their 4-1BB CAR under an inducible promoter allowing for transient expression and therefore killing. This approach demonstrated complete elimination of T-ALL xenograft tumors, but raised concerns about the clinical safety and the immunogenicity of transactivator proteins. Moreover, CD5 is not expressed by many malignant T cell clones and can be easily down regulated, potentially leading to antigen escape.

### CD7

CD7 is a transmembrane glycoprotein which is a primary marker for acute T-ALL and is highly expressed in a subset of T cell lymphomas (24, 30, 31). In normal tissues, CD7 expression is confined to T and natural killer (NK) cells. Recently, various groups have independently shown the potential of targeting CD7, however, all the studies reported a lack of CD7 downregulation on effector T-cells which resulted in extensive fratricide. Given the near universal expression of CD7 on normal T-cells, Gomes-Silva et al. used CRISPR/Cas9 system to disrupt the CD7 locus. Genetic knockout (KO) of CD7 led to normal expansion of CD7 specific CAR T cells without detectible fratricide of gene disrupted T cells. More importantly, they also demonstrated that anti-CD7 CAR T cells retained anti-viral activity *in vitro* which may provide protection in the context of T and NK ablation (13). These data led to the opening of a first in human phase I clinical trial (NCT03690011) of CD7 specific CAR-T cells in T cell leukemia and lymphoma.

A second group designed an elegant method to prevent membrane expression of CD7 protein called protein expression blocker (PEBL) by coupling an intracellular retention domain KDEL to an anti-CD7 single chain variable fragment. Transduction of anti-CD7 PEBL lead to abrogation of CD7 expression and inhibition of fratricide of PEBL CAR T cells. These modified T cells showed anti leukemic activity in cell-lines and patient derived xenograft (PDX) models of T-ALL (14). An additional advantage of this approach would be the possible direct translation to the clinic using existing current good manufacturing practice (cGMP) manufacturing processes.

Finally, Cooper and colleagues designed the first allogeneic CAR T cell product for the treatment of CD7 positive T cell tumors (15). They developed a CRISPR/Cas9 multiplex system targeting the T cell receptor alpha chain (TRAC) and CD7 genes. These UCART7 T cells were fratricide-resistant and killed PDX model of T-ALL *in vivo* without inducing xenogeneic graft-vs.-host disease (GvHD).

### CD30

CD30 is a more selectively expressed T lineage marker, present in all anaplastic large cell lymphomas (ALCLs) and some T-ALL (32, 33). Antibody drug conjugates such as brentuximab vedotin have been used to treat cutaneous lymphomas and advanced stage mycosis fungoides with clinical success (34, 35). Despite this success, significant drug conjugate mediated toxicities, including neuropathy (62%) and cytopenias (upwards of 78%), as well as limited durability of response, have led to the development of anti-CD30 CAR T cells. Although the majority of CD30. CAR-T cell trials have focused on Hodgkin lymphomas, results of 2 ALCL patients treated with CD30.CAR-Ts are promising and include one complete remission for 9 months without impaired virus-specific immunity (16).

### CCR4

CCR4 is a chemokine receptor abundantly expressed on the surface of T cell tumors including T-ALL, PTCL, and CTCL and is responsible for the homing of malignant cells to the skin. Various anti-CCR4 monoclonal antibodies are in development including mogamulizumab which already has approval in Japan for the treatment of relapsed/refractory adult T-cell leukemia/lymphoma. CCR4 has been validated as a CAR-T target as Perera and colleagues have demonstrated the ability of an anti-CCR4-CAR to lyse multiple T-cell lines *in vitro* and clear T-ALL tumor in a xenograft mouse model (18). Furthermore, since CCR4 is also expressed by T regulatory (Tregs) cells, the authors speculate that anti-CCR4 CAR T cells would be able to eliminate Tregs cells which have typically an immunosuppressive role in the tumor microenvironment. Nevertheless, given the wide expression of CCR4, safety questions about the toxicity profile of these cells still need to be addressed.

### CD37

CD37 is a four-passage transmembrane protein belonging to the tetraspanin superfamily. Our group and others have shown that it is expressed not only on B-cell tumors but on T cell malignancies as well (17). We generated CAR37 T cells and demonstrated specific *in vitro* killing of T cell lines, including HUT78 cells. We did not detect significant fratricide of CAR+ and/or untransduced T-cells *in vitro* lessening the concern for CAR-T mediated T cell aplasia. *In vivo* experiments and an upcoming phase I trial of CAR-37 will further evaluate CD37 as a therapeutic target.

### TRBC1 and TRBC2

Another promising approach is the targeting of the T-cell receptor beta-chain constant domain, type 1(TRBC1) or 2 (TRBC2). Maciocia and colleagues showed that T-cell malignancies are clonally restricted to either TRBC1 or TRBC2, akin to kappa/lambda clonality in multiple myeloma. They subsequently developed anti-TRBC1 CAR T cells and demonstrated specific killing of TRBC1 expressing normal and malignant T cells while sparing TRBC2 restricted cells (19). Given the near 50:50 distribution of TRBC1:TRBC2 expression, this approach offers the potential to spare an adequate number of normal T cells to maintain polyclonal T-cell immunity. An ongoing phase I/II clinical trial is evaluating the safety and efficacy of TRBC1 specific CAR-T cells in TRBC1 positive malignancies (NCT03590574). This group is also developing a novel TRBC2 binder and anti-TRBC2 CAR for TRBC2 restricted clones (20). These strategies open new opportunities for the treatment of T cell malignancies.

## CAR NK Cells Against T Cell Antigens

There is an expanding interest in using natural killer cells as source of autologous and allogeneic cellular therapy. NK cells are cytotoxic immune cells which represent our first line of defense against pathogens and malignant cells. NK cells express CD56, CD16, and CD7 but lack TCR, CD3, and CD5 expression (36). Notably, they are able to kill target cells in a non-antigen dependent fashion, do not cause GVHD and are short-lived relative to their T-cell counterparts. All these characteristics make them attractive candidates for genetic engineering with CAR molecules, especially in the context of T cell malignancies. As NK cells do not share as many common T-cell antigens, fratricide would not be expected during cellular manufacturing. Additionally, their short lifespan may prevent long-term T cell aplasia while their lack of a of TCR make them a potential source of allogeneic products.

For these reasons CAR NK cells targeting CD3, CD4, CD5, and CD7 have been explored (Table 1). Chen et al. utilized a NK-92 human cell line transduced with a 3rd generation anti-CD3 CAR and demonstrated *in vitro* activity against T-ALL cell lines and primary PTCL samples. Although these NK-92 CAR cells were unable to completely eradicate leukemic cells in a xenograft mouse model, improvements *in vivo* persistence may improve on the short lived nature of CD3CAR NK-92 cells (21). This same approach utilizing the NK-92 cell line for CAR therapy has been demonstrated for CD4 and CD5 positive lymphomas as well as with improvements in both *in vivo* persistence as well as activity in other disease such as T-ALL and PTCL (22, 23). Although NK-92 CARs carry promise, there is a significant safety concern regarding the use of a transformed human NK lymphoma cell line as an NK cell source. Approaches utilizing irradiated CAR NK-92 cells are being evaluated in clinical trials (NCT03081910, NCT02742727), however any approach meant to limit the persistence and expansion of an immortalized cell line *in vivo* will have similarly detrimental effects on CAR persistence and efficacy.

## Conclusions

The major challenges in the development of adoptive cell therapy for T cell tumors, as mentioned above, remain fratricide, T cell aplasia and the potential for leukemic transduction or poor T cell function if used in the autologous setting. Approaches to overcome fratricide include the genetic modification and deletion of the T cell antigen in the case of long-term CAR-T cell persistence or regulated CAR-T expression. To ensure restoration of T cell immunity, transient CAR expression can be achieved incorporation of a CAR suicide gene, transient CAR expression using mRNA electroporation, or short-lived NK cell lines. Finally, given that these toxicities may be tolerable initially, CAR-T cells followed by an ablative hematopoietic stem cell transplant may allow for hematologic rescue following CAR-T mediated disease clearance. As most of the models to date have utilized normal donor human T cells for CAR manufacturing, we must also consider the underlying fitness of the starting cell product. Peripheral T-cells from patients with underlying T cell malignancy routinely demonstrate impairments in T cell function as well as reduced quantities in peripheral blood, given the extensive prior treatment burden and immune-dysregulation. Approaches utilizing allogeneic donors and gene-editing techniques to remove the endogenous TCR, or CAR products generated from autologous or allogeneic NK cells may offer creative solutions. These include the potential for multiple allogeneic sources, such as peripheral blood, umbilical cord blood, or effector cells generated from induced pluripotent stem cells (iPCSs). Regardless of cell source, target antigen, and the challenges and obstacles each approach may carry, CAR-effector cells as a treatment option for T-cell lymphomas may provide an exciting opportunity for these diseases.

## Author Contributions

IS wrote and edited the manuscript. MF and MM contributed to manuscript planning and editing.

### Conflict of Interest Statement

The authors declare that the research was conducted in the absence of any commercial or financial relationships that could be construed as a potential conflict of interest.
